# Cytosolic and ER J-domains of mammalian and parasitic origin can functionally interact with DnaK

**DOI:** 10.1016/j.biocel.2006.11.006

**Published:** 2007

**Authors:** W.S. Nicoll, M. Botha, C. McNamara, M. Schlange, E.-R. Pesce, A. Boshoff, M.H. Ludewig, R. Zimmermann, M.E. Cheetham, J.P. Chapple, G.L. Blatch

**Affiliations:** a Department of Biochemistry, Microbiology and Biotechnology, Rhodes University, Grahamstown 6140, South Africa; b Department of Medical Biochemistry & Molecular Biology, Universität des Saarlandes, Homburg D66421, Germany; c Division of Molecular and Cellular Neuroscience, Institute of Ophthalmology, University College London, London EC1V 9EL, UK; d Center for Endocrinology, William Harvey Research Institute, Barts and the London, Queen Mary University of London, London C1M 6BQ, UK

**Keywords:** DnaJ, Malarial Hsp40s, Trypanosomal Hsp40s, Endoplasmic reticulum Hsp40s, HSJ1

## Abstract

Both prokaryotic and eukaryotic cells contain multiple heat shock protein 40 (Hsp40) and heat shock protein 70 (Hsp70) proteins, which cooperate as molecular chaperones to ensure fidelity at all stages of protein biogenesis. The Hsp40 signature domain, the J-domain, is required for binding of an Hsp40 to a partner Hsp70, and may also play a role in the specificity of the association. Through the creation of chimeric Hsp40 proteins by the replacement of the J-domain of a prokaryotic Hsp40 (DnaJ), we have tested the functional equivalence of J-domains from a number of divergent Hsp40s of mammalian and parasitic origin (malarial Pfj1 and Pfj4, trypanosomal Tcj3, human ERj3, ERj5, and Hsj1, and murine ERj1). An in vivo functional assay was used to test the functionality of the chimeric proteins on the basis of their ability to reverse the thermosensitivity of a *dnaJ cbpA* mutant *Escherichia coli* strain (OD259). The Hsp40 chimeras containing J-domains originating from soluble (cytosolic or endoplasmic reticulum (ER)-lumenal) Hsp40s were able to reverse the thermosensitivity of *E. coli* OD259. In all cases, modified derivatives of these chimeric proteins containing an His to Gln substitution in the HPD motif of the J-domain were unable to reverse the thermosensitivity of *E. coli* OD259. This suggested that these J-domains exerted their in vivo functionality through a specific interaction with *E. coli* Hsp70, DnaK. Interestingly, a Hsp40 chimera containing the J-domain of ERj1, an integral membrane-bound ER Hsp40, was unable to reverse the thermosensitivity of *E. coli* OD259, suggesting that this J-domain was unable to functionally interact with DnaK. Substitutions of conserved amino acid residues and motifs were made in all four helices (I–IV) and the loop regions of the J-domains, and the modified chimeric Hsp40s were tested for functionality using the in vivo assay. Substitution of a highly conserved basic residue in helix II of the J-domain was found to disrupt in vivo functionality for all the J-domains tested. We propose that helix II and the HPD motif of the J-domain represent the fundamental elements of a binding surface required for the interaction of Hsp40s with Hsp70s, and that this surface has been conserved in mammalian, parasitic and bacterial systems.

## 1 Introduction

While many classes of molecular chaperones exist, members of the heat shock protein 40 (Hsp40) and heat shock protein 70 (Hsp70) families form chaperone pairs that are amongst the most ubiquitous (Fink, 1999). The diverse cellular processes involving these chaperones include the correct folding of nascent polypeptide chains, prevention of protein denaturation and misfolding during cellular stress, degradation of proteins, protein translocation, and quaternary assembly/disassembly (Hennessy, Nicoll, Zimmermann, Cheetham, & Blatch, 2005b).

The major molecular chaperone, Hsp70, consists of an N-terminal ATPase domain and a C-terminal substrate-binding domain. The affinity of Hsp70 for protein client is modulated by ATP binding and hydrolysis. In the ATP bound state, affinity of the substrate-binding domain for the client protein is low and exchange rates are high. Hydrolysis of ATP to ADP results in high affinity for the substrate and low exchange rates, effectively locking the substrate into the binding pocket (Schmid, Baici, Gehring, & Christen, 1994). This integral step in assisted protein folding is directly modulated by the binding of Hsp40 proteins (Cheetham, Jackson, & Anderton, 1994; Liberek, Marszalek, Ang, Georgopoulos, & Zylicz, 1991). Furthermore, there is evidence that some Hsp40 proteins bind client protein first and then target client to Hsp70 (Han & Christen, 2003; Suh, Lu, & Gross, 1999).

Hsp40s are defined by the presence of an approximately 70 amino acid region known as the J-domain, which is essential for interaction with Hsp70. The J-domain is a highly conserved α-helical structure that interacts with the Hsp70 ATPase domain and possibly also with the Hsp70 substrate-binding domain (Auger & Roudier, 1997; Hennessy et al., 2005b; Suh et al., 1999). Hsp40s are divided into three groups based on their possession of domains in addition to the J-domain (Cheetham & Caplan, 1998). Type I Hsp40s contain four primary domains: an N-terminal J-domain, a glycine/phenylalanine (GF)-rich region, a zinc finger domain and a C-terminal domain. Type I Hsp40s have been shown to bind protein substrates at their C-terminal domain and to have independent chaperone activity by inhibiting denaturation and aggregation (Langer et al., 1992; Rüdiger, Schneider-Mergener, & Bukau, 2001). Type II Hsp40s contain an N-terminal J-domain, a GF-rich region and a C-terminal domain. Type III Hsp40s contain the J-domain, and this may occur at any position within the protein. Other than the J-domain, the type III Hsp40s are highly divergent in size, sequence and structure and tend to serve highly specialized functions. Specific Hsp40-Hsp70 partnerships have been identified that are dedicated to the correct folding of distinct subsets of client proteins. We and others have proposed that the J-domain makes important contributions to the affinity and specificity of binding of a specific Hsp40 protein to its partner Hsp70 (Garimella et al., 2006; Hennessy et al., 2005b).

The structures of the J-domain from six Hsp40 and Hsp40-like proteins have been published: *E. coli* DnaJ (Huang, Flanagan, & Prestegard, 1998; Pellecchia, Szyperski, Wall, Georgopoulos, & Wüthrich, 1996); human HDJ1 (Qian, Patel, Hartl, & McColl, 1996); *E. coli* Hsc20 (Cupp-Vickery & Vickery, 2000); the large T antigen from murine polyomavirus (Berjanskii et al., 2000); the large T antigen from SV40 in conjunction with the retinoblastoma tumour suppressor (Kim, Ahn, & Cho, 2001); and bovine auxilin (Gruschus, Greene, Eisenberg, & Ferretti, 2004; Jiang et al., 2003). The J-domain structures reveal the presence of four α-helices (helices I–IV) and a loop region between helices II and III. Helix I is usually seen as a short helix in type I Hsp40s. However, although there are a number of highly conserved hydrophobic residues in helix I, the tertiary structure of helix I, as seen from X-ray and NMR studies, becomes divergent in types II and III Hsp40s. The helices II and III are structurally conserved in all known J-domains, in particular helix II which bears an overall positive charge and is thought to interact with the negatively charged underside of the ATPase domain of Hsp70. Of particular importance is the HPD tripeptide that resides in the transhelix loop between helices II and III. Alteration of these residues always results in loss of functional interaction between Hsp40 and Hsp70 (Genevaux, Wawrzynow, Zylicz, Georgopoulos, & Kelley, 2001; Laufen et al., 1999; Mayer, Laufen, Paal, McCarty, & Bukau, 1999; Tsai & Douglas, 1996; Wittung-Stafshede, Guidry, Horne, & Landry, 2003). Apart from the HPD motif, the other amino acids on the J-domain of Hsp40 proteins that are involved in the binding to a partner Hsp70 are less precisely defined. However, as a result of our work (Hennessy, Cheetham, Dirr, & Blatch, 2000; Hennessy, Boshoff, & Blatch, 2005a) and that of other researchers (Garimella et al., 2006; Genevaux, Schwager, Georgopoulos, & Kelley, 2002; Genevaux et al., 2003; Lu & Cyr, 1998; Suh et al., 1999), other residues and regions outside the HPD motif, especially helices II, III and IV, are gradually being implicated in the general binding and specificity of interaction of Hsp40 proteins with Hsp70 proteins.

The high resolution structure of the *E. coli* DnaJ J-domain suggested that J-domain stabilization occurred through a buried core of hydrophobic residues, primarily Ile9, Leu10, Val12, Ile21, Ala53 and Leu57 (Hill, Flanagan, & Prestegard, 1995; Pellecchia et al., 1996; Szyperski, Pellecchia, Wall, Georgopoulos, & Wuthrich, 1994). Amino acid sequence alignment analysis of over 200 type I Hsp40s showed that Tyr7, Ala53 and Leu57 were conserved in over 98% of all sequences, and that Leu10 was absolutely conserved in all sequences (Hennessy et al., 2000). Tyr7 projects upward in the *E. coli* DnaJ J-domain tertiary structure and potentially makes contacts with residues of helices II, III and IV. Leu10 projects toward the helix II–helix III inter-helical space, potentially interacting with residues of helices II and III. Therefore, Tyr7 and Leu10 may be critical in ensuring the stability of the helix-loop structure of helix II–helix III for presentation to Hsp70 (Hennessy et al., 2005a). Arg26 has been shown to be critical for J-domain function in *E. coli* DnaJ and *Agrobacterium tumefaciens* DnaJ (Agt DnaJ), and has been proposed to be part of a network of residues on helix II (and possibly helix III) that form an Hsp70-binding site on the J-domain (Genevaux et al., 2002; Hennessy et al., 2005a,b). In addition, Genevaux et al. (2002) described Tyr25 of *E. coli* DnaJ as a candidate catalytic residue that potentially comprises part of this binding site. His33 of the HPD motif in the loop region has been extensively documented as being a critical residue in regulation of Hsp70 ATPase stimulation, and substitution of this residue results in loss of functional interaction between Hsp40 and Hsp70 (Genevaux et al., 2002; Kelley & Georgopoulos, 1997; Laufen et al., 1999; Mayer et al., 1999; Tsai and Douglas, 1996). Genevaux et al. (2002) revealed that a glycine substitution at Arg36 (Lys36 in Agt DnaJ) resulted in loss of J-domain function and suggested that the critical cluster for J-domain function is not only the HPD tripeptide but could also include Arg36 and Asn37 to form the critical HPD-R/K-N pentapeptide. Helix III appears to contain a so-called KFK motif (Hennessy et al., 2000), and a number of amino acid substitutions have been conducted on this motif. In particular, the F47A substitution in the J-domain of *E. coli* DnaJ resulted in loss of in vivo function (Genevaux et al., 2002), suggesting this was an essential amino acid for J-domain structure and function, potentially by forming interactions with His33 of the HPD motif. Interestingly, an F47L substitution in Agt DnaJ had no detectable effect on its in vivo function, suggesting that the Leu residue could sufficiently contribute to the contacts originally made by the Phe residue (Hennessy et al., 2005a). The highly conserved Leu57 of helix III projects into the J-domain interior and is likely to be a key residue in holding helices II and III together. Furthermore, experimental evidence has suggested that Leu57 was essential for J-domain function (Hennessy et al., 2005a). While residues of helices II and III and the loop region linking the two helices are crucial to J-domain function (Pellecchia et al., 1996), it has been proposed that the residues of helix IV are not essential to the co-chaperone function of DnaJ (Genevaux et al., 2002). However, recent studies involving the J-domain of Agt DnaJ identified residues on helix IV that were important for its in vivo function, suggesting a structural or functional role of this helix in other DnaJ homologues, potentially in the enhancement of the affinity or specificity of interactions with Hsp70 (Hennessy et al., 2005a,b). Asp59 and Arg63 are part of a conserved charged cluster of residues in helix IV, with Arg63 being part of the conserved QKRAA motif on helix IV of the J-domain of DnaJ. Data from studies on *E. coli* DnaJ (Suh et al., 1999) and Agt DnaJ (Hennessy et al., 2005a) suggested that substitutions of Asp59 and Arg63 partially disrupted the structure and function of these proteins, and recently it has been suggested that helix IV may contribute to the specificity of J-domains for their Hsp70 partners (Garimella et al., 2006; Hennessy et al., 2005b).

Hsp40 proteins are not completely interchangeable with respect to their interaction with distinct Hsp70s. The cytosolic Hsp70s, Hsc70, yeast Ssa1 and *E. coli* DnaK, are not interchangeable with BiP with respect to protein translocation into the endoplasmic reticulum (Brodsky, Hamamoto, Feldheim, & Schekman, 1993; Wiech, Buchner, Zimmermann, Zimmermann, & Jakob, 1993). Furthermore, *E. coli* DnaJ is capable of stimulating the ATPase activity of mammalian Hsc70, whereas mammalian Hdj1 is incapable of stimulating the ATPase activity of DnaK (Minami, Höhfeld, Ohtsuka, & Hartl, 1996). Hence, the J-domain may contain sequence and structural features that mediate the specificity of binding between Hsp40s and partner Hsp70s, and a number of J-domain swapping experiments have been conducted to establish the elements of specificity (reviewed in Hennessy et al., 2005b). In general, J-domains appear to be interchangeable when they are derived from Hsp40 proteins that interact with functionally equivalent or homologous Hsp70 proteins, or are involved in similar cellular processes (Deloche, Kelley, & Georgopoulos, 1997; Genevaux et al., 2001). However, J-domains appear to be less interchangeable when derived from Hsp40 proteins that are involved in very different cellular processes; for example, interchanging J-domains of type I Hsp40s with those of membrane-bound Hsp40s (Schlenstedt, Harris, Risse, Lill, & Silver, 1995) or viral Hsp40-like proteins such as the T antigen (Kelley and Georgopoulos, 1997; Sullivan et al., 2000). This suggests that two broad classes of J-domains may have evolved; those J-domains that have evolved to specifically interact with Hsp70s involved in assisted protein folding, and those J-domains that have evolved to specifically interact with Hsp70s involved in more specialized cellular processes. To date no systematic analysis has been conducted on the interchangeability of J-domains between all the types I, II and III Hsp40-like proteins from any one cell type, compartment or system.

In this study we have conducted domain swapping of the J-domains from a selection of divergent Hsp40s of mammalian and parasitic origin (malarial Pfj1 and Pfj4, trypanosomal Tcj3, human ERj3, ERj5, and Hsj1, and murine ERj1) in an attempt to identify system specific and common factors in Hsp40–Hsp70 interactions. The similarities and differences in the structure and function of Hsp40s of parasites and their hosts have yet to be determined, and therefore from this broader perspective we were interested in a comparative analysis of the J-domains of parasitic and human origin. An in vivo functional assay was used to assess the ability of the J-domains to substitute for the J-domain of a prokaryotic type I Hsp40. Furthermore, the functional importance of specific residues was addressed through single amino acid substitution analysis. The data suggested that cytosolic and ER J-domains of mammalian and parasitic origin can interact with DnaK using a common mechanism, and that a fundamental binding surface appears to be conserved in J-domains of Hsp40s of mammalian, parasitic and bacterial origin.

## 2 Materials and methods

### 2.1 Materials

*E. coli* OD259 (MC4100 *araD139* Δ*ara714* Δ*cbpA*::*kan dnaJ*::Tn10-42) was kindly provided by Dr. Olivier Deloche (University of Geneva, Switzerland). The pGEX-4T-ERj1, pGEX-4T-ERj3 and pGEX-4T-ERj5 plasmids encode mouse ERj1, human ERj3 and human ERj5. The pET23b-Tcj3 construct encoding Tcj3 has been described (Edkins, Ludewig, & Blatch, 2004), while the pCMV-Tag3a(Hsj1a) construct encodes Hsj1a. Mutagenesis was performed using the QuikChange site directed mutagenesis kit (Stratagene, USA) as per the manufacturer's instructions. Mutagenesis and PCR primers were synthesised by IDT (USA) and Inqaba Biotec (SA).

### 2.2 Creation of the Agt DnaJ chimera proteins

**Fig. 1:**
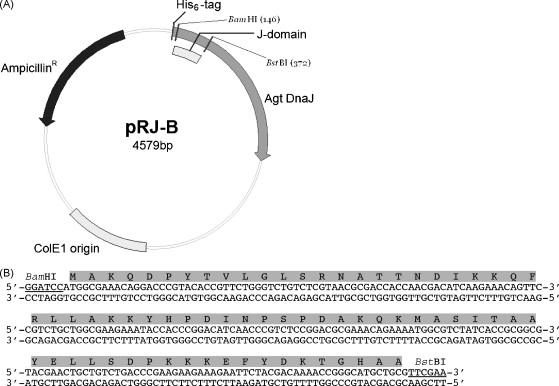
Plasmid pRJ-B used for deriving the constructs encoding Agt DnaJ chimeric proteins. (A) A plasmid map of pRJ-B: a *Bst*BI restriction site was engineered into the pRJ30 plasmid between the coding regions for the Agt DnaJ J-domain and GF region by silent mutation of the codon for Phe74 to produce the pRJ-B plasmid. The positions of the *Bam*HI and *Bst*BI restriction endonuclease sites used for swapping the J-domain coding regions in the creation of the Agt DnaJ chimeric proteins are indicated. (B) Codon optimised Pfj1 J-domain coding sequence. The translated J-domain protein sequence is highlighted and the *Bam*HI and *Bst*BI restriction endonuclease sites used for insertion of the coding region for the J-domain into the pRJ-B plasmid are underlined.

The pQE30-derived pRJ30 vector containing the Agt DnaJ coding sequence (Hennessy et al., 2005a) served as a base vector for all domain swapping and subsequent mutagenesis. A silent mutation encoding a *Bst*BI restriction site was introduced directly downstream of the J-domain at residue Phe74 of Agt DnaJ to produce vector pRJ-B (Fig. 1A). Removal of the coding region of the Agt DnaJ J-domain and insertion of the respective coding regions of the J-domains under investigation into the Agt DnaJ coding region backbone was achieved through use of the *Bst*BI restriction site and a *Bam*HI restriction site immediately upstream of the start of Agt DnaJ coding region (Fig. 1A).

*E. coli* codon optimized versions of the *Plasmodium falciparum* Pfj1 J-domain (Fig. 1B) and full length Pfj4 (Nicoll et al., 2006) were synthesized using Polymerase Chain Reaction (PCR) assembly (Stemmer, Crameri, Ha, Brennan, & Heyneker, 1995). The gene design process was also utilized to introduce the *Bam*HI and *Bst*BI sites to allow subsequent domain swapping. Subcloning of the coding regions for the Pfj1 (residues 60–128) and Pfj4 (residues 1–79) J-domains into the pRJ-B vector resulted in the creation of the expression constructs encoding the chimera Pfj1-J-Agt-DnaJ and Pfj4-J-Agt-DnaJ, respectively. The coding region for the Tcj3 (residues 1–74) J-domain was amplified by PCR from pET23b-Tcj3 with *Bam*HI and *Bst*BI restriction sites to allow subcloning into pRJ-B. Insertion of the coding region for the Tcj3 J-domain into the pRJ-B vector resulted in the creation of the expression construct encoding the Tcj3-J-Agt-DnaJ chimera. Similarly, the coding regions for the J-domains of Hsj1a (residues 1–72), ERj1 (residues 56–128), ERj3 (residues 23–97) and ERj5 (residues 35–100) were PCR amplified from pCMV-Tag3a (Hsj1a), pGEX-4T-ERj1, pGEX-4T-ERj3 and pGEX-4T-ERj5, respectively, and subcloned into pRJ-B to give expression constructs encoding Hsj1-J-Agt-DnaJ, ERj1-J-Agt-DnaJ, ERj3-J-Agt-DnaJ and ERj5-J-Agt-DnaJ. All mutations were produced by the whole-plasmid linear amplification approach using complementary oligonucleotides (QuikChange site directed mutagenesis kit, Stratagene, USA). Primers were designed so as to introduce or eliminate a restriction endonuclease site to facilitate the identification of successful mutants by restriction analysis. Mutations were confirmed by subsequent DNA sequencing.

### 2.3 In vivo complementation assays

Complementation assays were performed in the thermosensitive *E. coli dnaJ cbpA* strain, OD259 (Deloche et al., 1997; Kelley and Georgopoulos, 1997). Agt DnaJ has been shown to functionally replace CbpA and DnaJ in *E. coli* OD259 at 40 °C similarly to *E. coli* DnaJ (Hennessy et al., 2005a). Thus *E. coli* OD259 cells exogenously producing Agt DnaJ from a pQE30-based plasmid served as the positive control for the functional in vivo assays. Substitution of His33 of the HPD motif of Agt DnaJ is known to abolish interactions of the protein with DnaK (Hennessy et al., 2005a), therefore *E. coli* OD259 cells exogenously producing Agt DnaJ-H33Q from a pQE30-based plasmid served as the negative control.

Plasmids were transformed into competent *E. coli* OD259, and single colonies were used to inoculate 5 ml yeast-tryptone (YT) broth containing 100 μg/ml ampicillin for plasmid selection and 50 μg/ml kanamycin for strain selection. The cultures were grown overnight at 30 °C, before being diluted 1:100 with YT broth containing 100 μg/ml ampicillin and 50 μg/ml kanamycin, and grown further at 30 °C until an A_600_ of approximately 2.0 was reached. Cultures were diluted to an A_600_ of 0.3 and serial dilutions were performed to a final dilution of 1 × 10^−8^. Aliquots (3 μl) of each of these dilutions were spotted onto agar plates containing 50 μM IPTG. Plates were grown at 30 °C, 40 °C and 42 °C, respectively, to determine the ability of Agt DnaJ, Agt DnaJ-H33Q and the chimera proteins to reverse the thermosensitivity of *E. coli* OD259.

### 2.4 Western analysis for the detection of chimeras

Western analysis was performed on whole cell lysates of *E. coli* OD259 and its transformants producing His_6_-tagged Agt DnaJ and chimera proteins. Proteins were resolved on a 12% (acrylamide, w/v) sodium dodecyl sulphate polyacrylamide gel electrophoresis (SDS-PAGE) gel and transferred to nitrocellulose membrane. Proteins of interest were detected using a mouse anti-His antibody (Amersham, UK) and horseradish-peroxidase-conjugated anti-mouse secondary antibody (Amersham, UK) using chemiluminescence-based detection (ECL Western blotting kit, Amersham, UK). Images were captured using a Chemidoc chemiluminescence imaging system (Biorad, USA).

### 2.5 Binding studies with ERj1-J and BiP

The pGEX-4T-1-based construct containing the coding region for the J-domain of murine ERj1 (originally called Mtj1; Brightman, Blatch, & Zetter, 1995) fused downstream of the coding region for glutathione S-transferase (GST) has been described previously (Dudek et al., 2002). The heterologous overproduction and purification of the GST-ERj1-J fusion protein was carried out as described previously for other GST-J-domain fusion proteins (Tyedmers et al., 2000). Hamster BiP was generously provided by Dr. Martin Jung (Universität des Saarlandes, Germany). For the ERj1-BiP pull-down binding assays, purified GST-ERj1-J and its mutant derivatives were buffer exchanged into phosphate buffered saline (PBS) at 4 °C so as to remove GSH from the purified protein. A sample (80 μl) of a 50% slurry of GSH-Sepharose beads was equilibrated in PBS (200 μl). Sufficient GST-ERj1-J was added to the GSH-Sepharose suspension to give 0.5 μM final concentration. The suspension was incubated for 1 h at 4 °C to allow binding of GST-ERj1-J to the GSH-beads, before washing twice with PBS (300 μl). For the binding reaction, the bead-bound GST-ERj1-J was reconstituted in 200 μl of PBS, with and without ATP (2 mM), and with BiP (0.5 μM), and binding allowed to occur for 1 h at 4 °C. The beads were washed twice with PBS (200 μl), and the proteins eluted from the beads by treatment with SDS-PAGE sample treatment buffer (40 μl), and analysed by SDS-PAGE.

Surface plasmon resonance spectroscopy was carried out in a BIAlite upgrade system. Monoclonal goat anti-GST-antibodies (BIACORE, Uppsala, Sweden) were immobilized on a sensor chip CM5 (BIACORE, Uppsala, Sweden) by amine coupling according to the manufacturer's protocol. The sensor chip was equilibrated with running buffer (phosphate buffered saline containing 3 mM KCl, 1 mM MgCl_2_, 0.1% Tween 20 and 2 mM ATP). GST was bound to the immobilized antibodies in the reference cell, while the GST-ERj1-J fusion proteins were immobilized separately in the measuring cell (400 response units; flow rate 5 μl/min). Subsequently, solutions containing increasing concentrations of purified BiP (0.25–2 μM) were passed over the chip in the presence of ATP (flow rate 20 μl/min). Each BiP application was followed by the application of running buffer until baseline was reached. The analysis of the data were carried out using the BIAevaluation software version 2.2.4.

## 3 Results

### 3.1 Bioinformatic analysis of the J-domain and the identification of structurally and functionally important residues

**Fig. 2:**
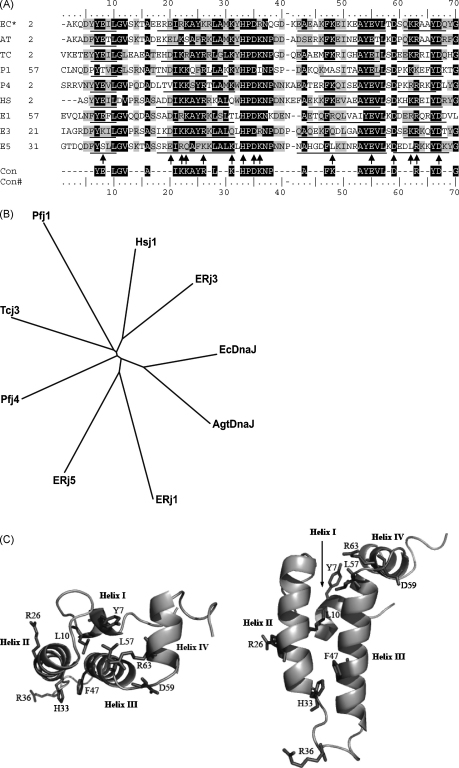
J-domains and conserved J-domain residues targeted for analysis. (A) Structure aided alignment of J-domains analyzed in this study. Predicted α-helices are underlined. Highly conserved charged residues are indicated by arrows. EC—*E. coli* DnaJ [GenBank accession number: P31689] residues 2–70; AT—*A. tumefaciens* DnaJ [AAR84666.1] residues 2–70; TC—*T. cruzi* Tcj3 [AAC18896] residues 2–71; P1—*P. falciparum* Pfj1 [NP_702750.1] residues 57–125; P4—*P. falciparum* Pfj4 [BAB17689] residues 2–71; HS—*Homo sapiens* HSJ1 [NP_001034639] residues 2–69; E1—*Mus musculus* ERj1 [NP_031895] residues 57–125; E3—*H. sapiens* ERj3 [NP_057390] residues 21–90; E5—*H. sapiens* ERj5 [NP_061854] residues 31–99; Con—consensus sequence of the aligned J-domain residues. Con#—consensus numbering used for all sequences in this paper, equivalent to the corresponding *E. coli* DnaJ residue numbering. *—Proteins of known structure. The numbers on the left-hand side of the alignment represent the positions of the first amino acid in each sequence. Sequences were aligned using ClustalW (Thompson, Higgins, & Gibson, 1994) and then adjusted by hand using available structural data. (B) The ClustalW alignment is represented as an unrooted radial tree using TreeView (Page, 1996). The protein names are indicated at the ends of the branches, and apart from *E. coli* DnaJ (EcDnaJ), the protein names are indicated by the standard abbreviations. (C) Locations of amino acid residues targeted for substitution shown on a ribbon representation of the known tertiary structure of *E. coli* DnaJ J-domain (PDB:1XBL). The structure is shown from two different orientations. Helices I–IV are indicated and the highlighted residues are shown as sticks and labelled using the single letter code. The ribbon representations of the structures were rendered using PyMol version 0.98 (DeLano, 2005).

The J-domain sequences analyzed in this work covered a wide range of diverse Hsp40 types (Fig. 2). Agt DnaJ, a prokaryotic type I Hsp40 that has 57% identity to the *E. coli* DnaJ, was previously shown to be able to reverse the thermosensitivity of a *dnaJ cbpA* mutant *E. coli* strain (OD259), suggesting that it was capable of functionally replacing DnaJ and CbpA (Hennessy et al., 2005a). Furthermore, Agt DnaJ-H33Q was unable to reverse the thermosensitivity of *E. coli* OD259, suggesting that Agt DnaJ reversed the thermosensitivity of *E. coli* OD259 through J-domain-based regulation of the chaperone activity of *E. coli* DnaK (Hennessy et al., 2005a). Due to the consistency and reproducibility of the results produced in functional in vivo complementation assays of *E. coli* OD259 producing Agt DnaJ, this protein was chosen as the type I Hsp40 backbone molecule for the creation of chimeras. It should be noted that J-domains chimeras were created using the *E. coli* DnaJ backbone, and the same complementation trends were observed as for the Agt DnaJ chimeras; however, the results were not as reproducible (data not shown). The lack of reproducibility of complementation assays using the *E. coli* DnaJ chimeras could possibly be related to the toxic effects that have been observed for the overproduction of *E. coli* DnaJ (Pröls et al., 2001). Tcj3 is a type I Hsp40 from *Trypanosoma cruzi*, the protozoan causative agent of Chagas’ disease (Edkins et al., 2004), while Pfj1 is a type I Hsp40 and Pfj4 is a type II Hsp40 identified from the protozoan malarial parasite *P. falciparum* (Watanabe, 1997). While no experimental evidence exists for the subcellular localization of Tcj3, the knowledge-based subcellular localization program PSORTII (Nakai & Kanehisa, 1992) predicted a cytosolic localization. Pfj1 has a potential mitochondrial import signal (RRKVCS; Watanabe, 1997), while Pfj4 was predicted to have a nuclear localization signal by PSORTII. HSJ1 is a human Hsp40 that is preferentially expressed in neuronal cells (Cheetham, Brion, & Anderton, 1992). Two forms of HSJ1 are found in vivo, both of which contain an identical J-domain. One form is localized to the cytosolic face of the ER (HSJ1b) while the other is cytosolic and nuclear (HSJ1a) (Chapple & Cheetham, 2003). ERj1 (also called Mtj1) is a mouse type III Hsp40 that has been shown to be enriched in microsomal and nuclear fractions of murine cells (Brightman et al., 1995). In particular it has been shown to be present in the endoplasmic reticulum (ER) in close association with active ribosomes (Dudek et al., 2002, 2005), and to interact with the ER Hsp70, BiP (Chevalier, Rhee, Elguindi, & Blond, 2000; Dudek et al., 2002). ERj3 (also called HEDJ) is a soluble Type I/II Hsp40 that has a Cys-rich region instead of a typical Cys-repeat region, and been shown to be present in the ER lumen and to functionally interact with BiP (Bies et al., 1999, 2004; Yu & Haslam, 2005). ERj5 (also called JPDI) is a type III Hsp40 that contains thioredoxin motifs characteristic of a protein disulfide isomerase (Cunnea et al., 2003; Hosoda, Kimata, Tsuru, & Konho, 2003). ERj5 is located in the ER lumen and has been shown to interact with BiP in an ATP-dependent manner (Cunnea et al., 2003).

The sequences of the J-domains used in this study were aligned, phylogenetically analysed, and key conserved residues identified and highlighted on the three-dimensional structure of the *E. coli* DnaJ J-domain (Fig. 2). We derived a J-domain consensus sequence for the highly conserved sequences, and also identified the positions of highly conserved charged residues (Fig. 2A). The numbering used for residues in the ensuing text will be the J-domain consensus numbering which is equivalent to the *E. coli* DnaJ J-domain numbering (Fig. 2A). All of the J-domain sequences exhibited conservation of most of the residues previously identified to be conserved from a study of over 200 J-domains (Hennessy et al., 2000). Phylogenetic tree analysis (Fig. 2B) revealed the relative relatedness of the J-domains of the prokaryotic Hsp40s (Agt DnaJ and *E. coli* DnaJ), certain parasitic Hsp40s (Pfj1 and Tcj3) and certain mammalian Hsp40s (HSJ1 and ERj3; ERj1 and ERj5). The J-domain of Pfj4 appeared to be relatively divergent from all the other J-domains. In this study, the amino acid residues targeted for substitution were chosen based on their conservation (Fig. 2A), predicted orientation in three dimensions (Fig. 2C), and predicted structural and functional roles as determined from previous studies on DnaJ from our group and others (Genevaux et al., 2002; Hennessy et al., 2005a).

### 3.2 Characterization of the chimeras

**Fig. 3:**
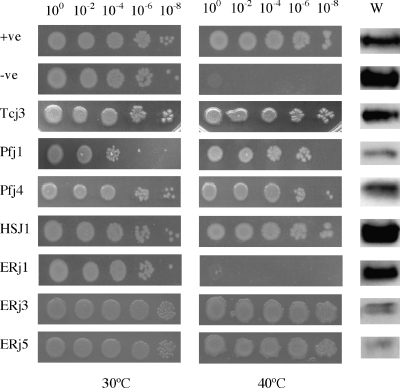
All the J-domain chimeras of Agt DnaJ, apart from ERj1-J-Agt-DnaJ, were able to reverse the thermosensitivity of *E. coli* OD259. Plasmids encoding each of the chimeric Hsp40s were transformed into the temperature sensitive strain *E. coli* OD259. Cells were diluted sequentially, spotted onto agar plates supplemented with IPTG and grown at the non-stress temperature of 30 °C and stress temperature of 40 °C. The dilution factor is indicated above each growth panel. The ability of each protein to compensate for the lack of DnaJ and CbpA was investigated by monitoring the reversal of thermosensitivity under stress temperature conditions at 40 °C. The proteins produced in the cells are indicated with an abbreviation on the left-hand side of each growth panel: +ve—Agt DnaJ; −ve—Agt DnaJ-H33Q; Tcj3—Tcj3-J-Agt-DnaJ; Pfj1—Pfj1-J-Agt-DnaJ; Pfj4—Pfj4-J-Agt-DnaJ; HSJ1—HSJ1-J-Agt-DnaJ; ERj1—ERj1-J-Agt-DnaJ; ERj3—ERj3-J-Agt-DnaJ; ERj5—ERj5-J-Agt-DnaJ. The levels of chimeric protein production in *E. coli* OD259 were determined by Western analysis (W).

Each of the Hsp40 J-domain chimera-encoding plasmid constructs were transformed into *E. coli* OD259, and the ability of the chimeras to reverse the thermosensitivity of this strain was assessed by comparing growth at 30 °C and 40 °C, respectively. For the *E. coli* OD259 cells producing Agt DnaJ (positive control), growth at 30 °C occurred up to the highest dilution (10^−8^), indicating that Agt DnaJ was not toxic to the cells. Furthermore, the growth profile observed at 40 °C was similar to that observed at 30 °C, suggesting that Agt DnaJ was able to reverse the thermosensitivity of *E. coli* OD259 (Fig. 3). This result was consistent with our previously published data that demonstrated Agt DnaJ was able to replace DnaJ and CbpA in *E. coli* OD259 and reverse the thermosensitivity of this strain (Hennessy et al., 2005a). As expected, *E. coli* OD259 producing Agt DnaJ-H33Q (negative control), was able to grow at 30 °C, but was unable to grow at 40 °C. *E. coli* OD259 transformants producing the chimeras HSJ1-J-Agt-DnaJ, Pfj1-J-Agt-DnaJ, Pfj4-J-Agt-DnaJ, Tcj3-J-Agt-DnaJ, ERj3-J-Agt-DnaJ and ERj5-J-Agt-DnaJ were all able to grow at 30 °C, and reversed the thermosensitivity of this strain at 40 °C similar to the control (Fig. 3). Furthermore, the H33Q mutant version of all these chimeras disrupted their in vivo functionality (Table 1 and data not shown). These data indicated that each of the J-domains were able to functionally replace the J-domain of Agt DnaJ in this prokaryotic system, and most likely exerted their in vivo function through a functional interaction with *E. coli* DnaK. The only chimera that was unable to reverse the thermosensitivity of *E. coli* OD259 was the ERj1-J-Agt-DnaJ. Western analysis to determine the protein production levels of the various chimeras in *E. coli* OD259, demonstrated that the chimeric proteins were produced and detectable in all the transformed strains (Fig. 3). This indicated that the inability of the ERj1-J-Agt-DnaJ chimera to reverse the thermosensitivity of *E. coli* OD259 was due to a lack of functionality rather than an absence of protein.

### 3.3 Pfj1, Pfj4 and Hsj1 J-domain mutants

The ability of this wide range of J-domains to functionally substitute for the Agt DnaJ J-domain in the *E. coli* system suggested that an underlying commonality existed in their mode of interaction with the *E. coli* DnaK. Therefore, a comparative analysis was conducted of the functional effects of the substitution of certain conserved amino acids of the J-domains of these proteins (Table 1).

Production of the helix I mutant protein Pfj4-J-Agt-DnaJ-Y7A in *E. coli* OD259 partially reversed its thermosensitivity indicating this protein was partially functional in this assay, while the Pfj4-J-Agt-DnaJ-L10A was unable to reverse the thermosensitivity of this strain indicating it was non-functional in this assay (Table 1). Similar results were observed for the equivalent helix I mutant proteins of HSJ1-J-Agt-DnaJ. The helix II mutant protein Pfj4-J-Agt-DnaJ-R26A was partially functional (Table 1), while the HSJ1-J-Agt-DnaJ-R26A, Pfj1-J-Agt-DnaJ-R26A, and Pfj1-J-Agt-DnaJ-F25A mutant proteins were all non-functional (Table 1).

As discussed in the previous section, the H33Q substitution in the loop HPD motif was found to disrupt the functionality of all the chimeras. By contrast, substitution of the basic residue of the so-called HPD-R/K-N pentapeptide in Pfj4-J-Agt-DnaJ-K36A, did not affect its functionality in the in vivo assay (Table 1).

The so called KFK motif of helix III is replaced by a KMA motif in the J-domain of Pfj1. Substitution of Met47 with Phe47 (the KMA to KFA mutation) resulted in a mutant Pfj1-J-Agt-DnaJ protein that was only partially functional in the in vivo assay (Table 1). Further substitution of the KMA motif of the Pfj1 J-domain to produce KMK and KFK, resulted in mutant Pfj1-J-Agt-DnaJ proteins that were fully functional. The RFK motif on helix III of the J-domain of Pfj4 was modified to RAK, and the resultant mutant protein Pfj4-J-Agt-DnaJ-F47A was found to be non-functional. Furthermore, substitution of the conserved helix III L57 residue in Pfj4-J-Agt-DnaJ-L57A resulted in a non-functional mutant protein (Table 1).

The roles of key conserved residues (e.g. D59) and motifs (e.g. the “QKRAA” motif) in helix IV were also investigated in this study (Table 1). The equivalent helix IV mutant proteins Pfj4-J-Agt-DnaJ-D59A and Hsj1-J-Agt-DnaJ-D59A were both found to be functional. The Pfj4-J-Agt-DnaJ-R63A mutant protein (Arg63 of the KR**R**RK motif of Helix IV in Pfj4, corresponding to the QK**R**AA motif of Agt DnaJ) was found to be functional, while in contrast the Pfj1-J-Agt-DnaJ-K63A mutant protein (substitution of Lys63 of the KK**K**EF motif of Helix IV in Pfj1) was only partially functional. Mutation of the Pfj1 KKKEF motif to the more conserved QKRAA motif produced a functional Pfj1-J-Agt-DnaJ mutant protein.

Production of all the mutant chimeric proteins was detected by Western analysis, suggesting that the inability of certain mutant proteins to reverse thermosensitivity was the result of a lack of protein functionality rather than a lack of protein production (Table 1). As has been previously observed (Hennessy et al., 2005a), levels of protein production in *E. coli* OD259 often vary between mutants and do not necessarily correlate with levels of functional recovery.

### 3.4 ERj1 J-domain

**Fig. 4:**
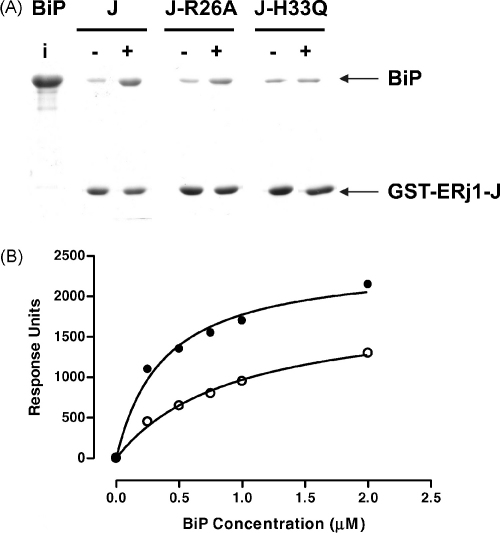
The R26A helix II mutation of the ERj1 J-domain disrupts binding to BiP, but not as extensively as the H33Q mutation of the HPD motif. (A) SDS-PAGE analysis of pull down assays conducted to determine the relative binding to BiP (0.5 μM) of equimolar concentrations of immobilized GST-ERj1-J (J), GST-ERj1-J-R26A (J-R26A) and GST-ERj1-J-H33Q (J-H33Q) in the presence (+) and absence (−) of ATP (2 mM). The input BiP is indicated (i) and the positions of co-purifying BiP and GST-ERj1-J and its derivatives are indicated by arrows. (B) Graphical presentation of the SPR data for ERj1-J (solid circles) and ERj1-J-R26A (open circles) binding to BiP. Four hundred response units of GST, GST-ERj1-J and GST-ERj1-J-R26A were bound to anti-GST antibodies covalently attached to a CM5 sensor chip. The GST fusion proteins were separately bound to the antibodies in the measuring cell, while the GST was bound to the antibodies in the reference cell. A number of different BiP solutions covering a range of concentrations (hamster BiP; 0.25, 0.75, 1.0 and 2.0 μM) were passed over the sensor chip in the presence of ATP. Each BiP application was followed by the application of running buffer containing ATP, thus allowing the association and dissociation kinetics to be followed. The maximum response units recorded for the association phase were plotted against concentration, and a curve fitted to the data by non-linear regression assuming one-site binding (hyperbola setting; GraphPad Prism version 4.00 for Windows, Graphpad Software, San Diego, California, USA, www.graphpad.com). The response units were recorded as the difference between the measuring and the reference cell. Note, the R26A and H33Q substitutions were numbered according to the consensus numbering; R82A and H89Q would be the actual numbering based on the ERj1 sequence.

Since it was not possible to characterize the ERj1 J-domain any further through an in vivo assay, an in vitro analysis was conducted. Using a GST-ERj1-J fusion protein and mutant derivatives, GST-ERj1-J-R26A and GST-ERj1-JH33Q, affinity pull-down assays were conducted to evaluate the interaction of the J-domains with BiP in the presence and absence of ATP (Fig. 4A). While GST-ERj1-J functionally interacted with BiP in an ATP-dependent manner, GST-ERj1-J-H33Q had no significant interaction with BiP since the levels of BiP detected in the pull-down assay were equivalent in the presence and absence of ATP. The GST-ERj1-J-R26A protein exhibited an ATP-dependent interaction with BiP, but at reduced levels compared to GST-ERj1-J. The interaction of GST-ERj1-J-R26A was further assessed using surface plasmon resonance spectroscopy (SPR; Fig. 4B). Using a range of BiP concentrations (0.25–2.0 μM), the apparent affinity of GST-ERj1-J-R26A for BiP was found to be reduced by 50% compared to the affinity of GST-ERj1-J for BiP (Fig. 4B). The results of the in vitro study on GST-ERj1-J-R26A were consistent with the results of the in vivo assays on mutant J-domain chimeras, where we have found that substitution of the equivalent residue at position 26 on helix II of other J-domains disrupted their functionality in the in vivo assay (J-domains of Pfj1, Pfj4 and HSJ1).

## 4 Discussion

Apart from the J-domain of the integral-membrane-bound ER Hsp40, ERj1, our findings suggested that all the J-domains tested exerted their functionality in the in vivo assay through a specific interaction with *E. coli* Hsp70, DnaK. Therefore, we propose that cytosolic and ER J-domains of mammalian and parasitic origin can interact with DnaK using a common mechanism. Furthermore, we found that substitution of a basic residue at position 26 of the helix II of the J-domain compromised functionality in all the J-domains in which this mutation was investigated (J-domains of Pfj1, Pfj4, HSJ1 and ERj1). Similar results have been found for *E. coli* DnaJ (Genevaux et al., 2002) and Agt DnaJ (Hennessy et al., 2005a,b). Therefore, we propose that this basic residue of helix II together with the HPD motif of the loop region are important elements of a fundamental binding surface required for J-domain-based Hsp40-Hsp70 interaction. This fundamental binding surface appears to be conserved in J-domains of Hsp40s of mammalian, parasitic and bacterial origin.

### 4.1 J-domain interchangeability

The number of different Hsp40 proteins in any organism generally outweighs the number of different Hsp70 proteins identified; for example, there are 6 Hsp40s and 3 Hsp70s in *E. coli* (Hennessy et al., 2005a), while there are 43 Hsp40s and 6 Hsp70s in *P. falciparum* (Matambo, Odunuga, Boshoff, & Blatch, 2004; Sargeant et al., 2006). This suggests the Hsp70 protein is the more promiscuous member of the Hsp40/Hsp70 pair and that the Hsp40 protein provides the specificity to this chaperone partnership. The J-domain may provide some of the molecular determinants of this specificity (Garimella et al., 2006; Hennessy et al., 2005a,b). In this study we have analyzed the J-domains from a number of diverse Hsp40s to assess their ability to functionally replace the J-domain of an exogenously produced Agt DnaJ in the prokaryotic *E. coli* OD259 in vivo complementation system. Interestingly, while Pfj1, Pfj4 and Tcj3 were all of parasitic origin and HSJ1, ERj3, and ERj5 were of mammalian origin, the J-domains of each protein were able to functionally replace the J-domain of the prokaryotic Agt DnaJ J-domain. In contrast, the J-domain of ERj1, the only integral membrane-bound Hsp40 tested, was unable to substitute for the J-domain of Agt DnaJ in the in vivo complementation assay. Two other membrane-bound ER Hsp40s, ERj2 and ERj4, have been investigated previously by others. Using a yeast complementation assay, Schlenstedt et al. (1995) found that the J-domains from the yeast Hsp40s Sis1p (cytosolic) and Mdj1p (mitochondrial lumen) were unable to functionally replace the J-domain of the integral membrane-bound Hsp40, Sec63p (ERj2 homologue). By contrast, the J-domain of the membrane-associated ERj4 (also called Mdg1) was shown to functionally replace the J-domain of *E. coli* DnaJ in an *E. coli* complementation assay (Pröls et al., 2001). Membrane-bound Hsp40s have also been investigated in prokaryotic systems. Kluck et al. (2002) showed that the J-domain of a membrane-bound *E. coli* Hsp40, DjLC, could not replace that of cytosolic *E. coli* DnaJ. This result may reflect that fact that DjLC was shown to interact with a specialized *E. coli* Hsp70 called HscC, which did not interact with *E. coli* DnaJ (Kluck et al., 2002). The lack of interaction of the J-domain of ERj1 with DnaK in the in vivo complementation assay, suggested that it may have reduced affinity for Hsp70s other than its partner BiP. It is well established that ERj1 can functionally interact with BiP, and that this association is important for the role of ERj1 in protein translocation into the ER (Chevalier et al., 2000; Dudek et al., 2002). It has also been shown that the ERj1 J-domain could stimulate *E. coli* DnaK ATPase activity in vitro, however, only at levels greater than that required for similar stimulation by *E. coli* DnaJ (Chevalier et al., 2000). This finding suggests that the ERj1 J-domain has low affinity and specificity for DnaK, and is consistent with our findings. It would be worthwhile conducting a gain-of-function analysis on the ERj1 J-domain by changing certain divergent residues of the ERj1 J-domain to those found in other J-domains (e.g. Phe9 to Ile or Leu), and evaluating which residues promote a functional interaction with DnaK using both in vitro and in vivo assays.

### 4.2 Targeted mutagenesis

#### 4.2.1 Helix I

Published J-domain structures have revealed that while helix IV is relatively mobile, helix I is relatively fixed in position (Berjanskii et al., 2000; Cupp-Vickery & Vickery, 1997, 2000; Huang et al., 1998; Pellecchia et al., 1996; Qian et al., 1996). The side-chains of the helix I residues Tyr7 and Leu10 may be involved in core contacts with helices I, II and III thereby stabilizing the J-domain scaffold for interactions with Hsp70 (Berjanskii et al., 2000). Substitution of these residues to Ala resulted in partial and complete reduction of J-domain functionality, respectively, in both the Pfj4-J-Agt-DnaJ and HSJ1-J-Agt-DnaJ chimeras. This was consistent with the findings of Hennessy et al. (2005a,b) where a lack of functionality was shown for Y7A and L10A mutant Agt DnaJ proteins in the same in vivo functional assay. These substitutions most likely destabilized the J-domain, resulting in a destabilized mutant protein with compromised functionality. Therefore, while these data suggest that Y7 and L10 of helix I have primarily a structural role, they do not exclude the possibility that these residues have an indirect functional role ensuring that the J-domain is correctly orientated for interactions with Hsp70 (Hennessy et al., 2005a).

#### 4.2.2 Helix II and the loop

Based on structural data from the J-domain of polyomavirus T antigen (Berjanskii et al., 2000), residues Tyr25 and Lys26 of the positively charged helix II of the J-domain of DnaJ were predicted to form part of the interaction surface that contacts the ATPase domain of DnaK. When substituted, these residues were found to disrupt in vivo functionality of *E. coli* DnaJ and Agt DnaJ (Genevaux et al., 2002; Hennessy et al., 2005a,b). Our findings were consistent with these data, in that the R26A mutations in the J-domains of Pfj1-J-Agt-DnaJ, Pfj4-J-Agt-DnaJ, HSJ1-Agt-DnaJ and GST-ERj1-J, and the Y25A mutation of the J-domain of Pfj1-J-Agt-DnaJ J-domain disrupted functionality. Lys36 (equivalent to Arg36 in *E .coli* DnaJ) is part of the functionally critical pentapeptide (HPD-R/KN) predicted by Genevaux et al. (2002). While it has been shown that a R36G mutation in *E. coli* DnaJ resulted in loss of function (Genevaux et al., 2002), we have demonstrated that a K36A mutation in Pfj4-J-Agt-DnaJ had no effect on its in vivo functionality. This suggested that while Arg36 appeared to be a structurally and/or functionally critical residue in *E. coli* DnaJ, it did not appear to be critical in all Hsp40s.

#### 4.2.3 Helix III

Phe47 of the KFK motif has been predicted to be important in J-domain function and was previously the only residue in close proximity to His33 that abolished Hsp40 function when mutated (Genevaux et al., 2002). Since Phe47 protrudes into the inter-helical space of helices II and III, it is tempting to propose that it has a largely structural role in maintaining the orientation of the J-domain and particularly the loop region. Indeed, it has been suggested that Phe47 may sterically constrain the movement of the helix II–helix III inter-helical loop (Genevaux et al., 2002); however, its conserved nature and proximity to His33 also suggest a potential mechanistic role in Hsp40–Hsp70 interactions (Hennessy et al., 2000; Landry, 2003). Here we have demonstrated that the mutation F47A in the Pfj4-J-Agt-DnaJ chimera completely abolished functionality of the chimera, as is seen for the equivalent mutation in *E. coli* DnaJ, yeast Ydj1 and an Hdj1 *E. coli* DnaJ chimera (Genevaux et al., 2002; Hennessy et al., 2005a,b; Johnson & Craig, 2000). While the function of Phe47 is unclear, these findings demonstrate that this residue is necessary for the functioning of certain diverse Hsp40s. Interestingly, the F47L substitution in Agt DnaJ had no adverse effect on its in vivo functionality (Hennessy et al., 2005a,b), suggesting that the minimum requirement for functionality at this position was the presence of a large hydrophobic residue. This was further supported by our data in which the M47F substitution of the KMA motif of the Pfj1-J-Agt-DnaJ chimera preserved functionality of the mutant protein, albeit at reduced levels. This was similarly the case when the neighbouring hydrophobic residue Ala48 was mutated to the more frequently encountered conserved residue Lys48, and when the double mutation M47F/A48K (producing a KFK motif) was performed on the Pfj1-J-Agt-DnaJ chimera. Leu57 is predicted to be a key residue involved in maintaining the structural integrity of helices II and III, since its side chain projects directly into the helix II–helix III cleft (Hennessy et al., 2005a,b). The L57A mutation of the Pfj4-J-Agt-DnaJ chimera, and the L57S mutation of Agt DnaJ (Hennessy et al., 2005a,b) abolished in vivo functionality of these Hsp40 proteins, suggesting that Leu57 was indeed required for the structure and function of the J-domain.

#### 4.2.4 Helix IV

Residue Asp59 is predicted to be of structural importance in the J-domain due to its location on the border of helices III and IV. Structural data suggests that helix IV is mobile, and its exact functional location is unclear. Hennessy et al. (2005a,b) showed that a D59A mutation of Agt DnaJ abolished its in vivo functionality; however since the mutant protein was undetectable, the inability of the protein to function in vivo may be attributed to a lack of protein production. Conversely, Genevaux et al. (2002) demonstrated that a T58A/D59A double mutation in *E. coli* DnaJ had no effect on its in vivo functionality. Our data showing that the Pfj4-J-Agt-DnaJ-D59A and Hsj1-J-Agt-DnaJ-D59A chimeras were functional in the in vivo assay, were consistent with the *E. coli* DnaJ data (Genevaux et al., 2002). Arg63 is part of the conserved QKRAA motif of helix IV that has been proposed to play a role in enhancing the affinity or specificity of interactions with Hsp70 (Garimella et al., 2006; Hennessy et al., 2005a,b). In this study, Pfj1-J-Agt-DnaJ-K63A was fully functional in the in vivo assay, and Pfj4-J-Agt-DnaJ-R63A was partially functional. While this result suggested that a basic residue at position 63 was not absolutely critical for J-domain-based interactions with Hsp70, it was consistent with its proposed role in the affinity or specificity of interaction.

### 4.3 Conclusion and future perspectives

This study has analysed cytosolic and ER Hsp40s of mammalian and parasitic origin, and found that certain key features of the J-domain appear to be fundamental to the function of all the J-domains studied, and perhaps to the function of J-domains in general. Interestingly, the differences appear to be subtle (e.g. the effects of the Y7A and R26A substitutions on the function of the J-domains of Hsj1 versus Pfj4), and may reflect slight differences in affinity or specificity of these J-domains for DnaK. These differences need to be probed further using quantitative in vitro assays, and incorporating an analysis of the less-conserved J-domain residues shown by NMR analysis to occur at J-domain-DnaK/Hsp70 binding interfaces. Furthermore, the possibility that there are specialized features unique to the J-domains of integral-membrane-bound Hsp40s needs to be investigated more extensively.

## Figures and Tables

**Table 1:**
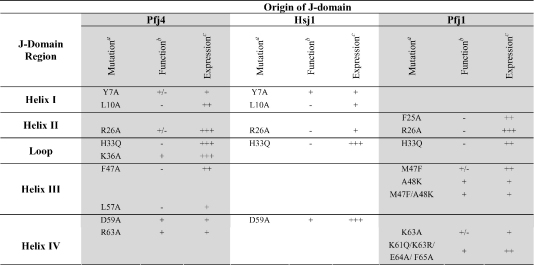
Functional analysis and expression levels of mutant J-domain chimera proteins

^a^Mutations were assigned according to the consensus sequence detailed in Fig. 2A. ^b^To assess the effect of the mutations on functionality of the J-domains, *E. coli* OD259 cells producing the mutant chimera proteins were analysed for their ability to grow under non-stress and stress temperatures: ‘+’—reversal of thermosensitivity (growth at 40 °C and at 42 °C); ‘−’—no reversal of thermosensitivity (no growth at 40 °C or 42 °C); or ‘±’—partial reversal of thermosensitivity (growth at 40 °C and less or no growth at 42 °C). ^c^Expression levels of the mutant proteins in *E. coli* OD259 were assessed by Western analysis: “+++’—higher levels than those observed for the positive control (Agt DnaJ); ‘++’—medium levels similar to those observed for Agt DnaJ; ‘+’—lower levels than those observed for Agt DnaJ.
